# Reduction behavior of chromium(VI) with oxalic acid in aqueous solution

**DOI:** 10.1038/s41598-020-74928-7

**Published:** 2020-10-20

**Authors:** Hao Peng, Jing Guo

**Affiliations:** Chongqing Key Laboratory of Inorganic Special Functional Materials, College of Chemistry and Chemical Engineering, Yangtze Normal University, Fuling, 408100 Chongqing People’s Republic of China

**Keywords:** Environmental sciences, Environmental chemistry, Pollution remediation

## Abstract

The direct Cr(VI) reduction process by oxalic acid was conducted. The existence of Cr(VI) in the reaction medium was measured by software Visual MINTEQ and the concentration of Cr(VI) was measured by ICP-OES. The results showed that the Cr(VI) was efficiently reduced by oxalic acid at high reaction temperature and high dosage of oxalic acid. The reduced product, Cr(III), was easily generated stable complex compounds (Cr(HC_2_O_4_)_3_) with oxalate, which displayed a negative effect on the reduction process. The high reaction temperature and high acidic medium could destroy the stable structure of a complex compound to release oxalate, and facilitate the reduction of Cr(VI). Generally, the results showed in this paper provided a versatile strategy for Cr(VI) reduction and exhibited a bright application future for real wastewater treatment.

## Introduction

Chromium(VI), placed in the fourth period of the periodic table^1–3^, was a toxic heavy metal and is classified in Group 1 (carcinogenic to humans) by the International Agency for Research on Cancer^4,5^. It was harmful to the environment and human health, thus, some treatments were needed.

Nowadays, many technologies had been developed for Cr(VI) removal from wastewater. All the technologies could be divided into three kinds: advanced oxidation technology, electrochemical technology and physicochemical technology^6^. Chemical precipitation, ion exchange, membrane filtration and adsorption belonged to physicochemical technologies, which were normal and easy to conduct^7–10^. The other mature technology associated with electricity was called electrochemical technology, which contained electrochemical reduction, electrocoagulation, electrodialysis and electrode-ionization, etc.^11–13^. Advanced oxidation technology contained photocatalysis and nanotechnology were practical approaches in treating wastewater^14,15^. It was needed to develop new efficient techniques for Cr(VI) removal as problems associated with causing secondary pollution, large scale application and high cost were remained in current technologies. Recently, reduction of Cr(VI) to Cr(III) had attracted significant attention^12,13,16,17^.

Oxalic acid was a widely used natural organic acid, which was mainly decomposed of plant root and organic matters^18^. It had been used to reduced Cr(VI) due to its environmental-friendly nature and low cost^19,20^. Mu investigated the reduction process of Cr(VI) with oxalic acid within and without Mn(II)^21^. The results showed that the Cr(VI) could not be reduced in the oxalic acid solution or Mn(II) solution, while nearly 99% Cr(VI) reduced to Cr(III) in the oxalic acid solution mixed with Mn(II). Thus, they concluded that Mn(II) could catalyze the reduction process of Cr(VI) with oxalic acid. Many methods had been developed to promote the reduction process of Cr(VI) with oxalic acid, like catalyzed by TiO_2_, Al_2_O_3_, FeOOH and sunlight^22–25^, but these methods were still not easy for the practical application, especially in groundwater remediation. This paper focused on the directional reduction behavior of Cr(VI) with oxalic acid because of the direct Cr(VI) reduction by oxalic acid was thermodynamically spontaneous^26–28^, the effect of dosage of oxalic acid, reaction temperature, dosage of sulfuric acid and reaction time on the reduction efficiency of Cr(VI) were investigated. Meanwhile, the reaction mechanism and reduction kinetics were conducted.

## Results and discussion

**Figure 1:**
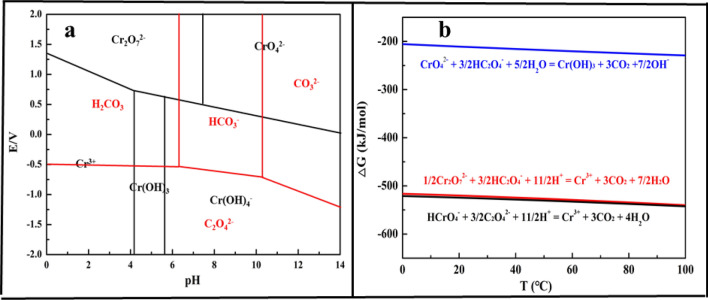
(**a**) E-pH diagram of Cr(VI) and oxalic acid at 25 °C; (**b**) Relationship between ΔG and temperature of reduction of Cr(VI).

Oxalic acid (H_2_C_2_O_4_) could be used as a reductant for Cr(VI) reduction as E^0^(HCrO_4_^−^/Cr^3+^) = 1.35 V and E^0^(C_2_O_4_^2−^/CO_2_) = 0.49 V^29^. With a summarization of the potential-pH diagram of chromium and oxalic acid shown in Fig. 1a, it was clear that the position of oxalic acid was always lower than Cr(VI), which indicated that the potential of oxalate was lower than Cr(VI). Therefore, oxalic acid could reduce Cr(VI) into Cr(III). The main reaction during the reduction process was between Cr(VI) and oxalic acid. The ΔG of main reactions were calculated at 25 °C^30–32^. The results displayed in Fig. 1b showed that the ΔG was negative, which indicated that the reduction of Cr(VI) with oxalic acid was thermodynamically spontaneous.

### Reaction mechanism

**Figure 2:**
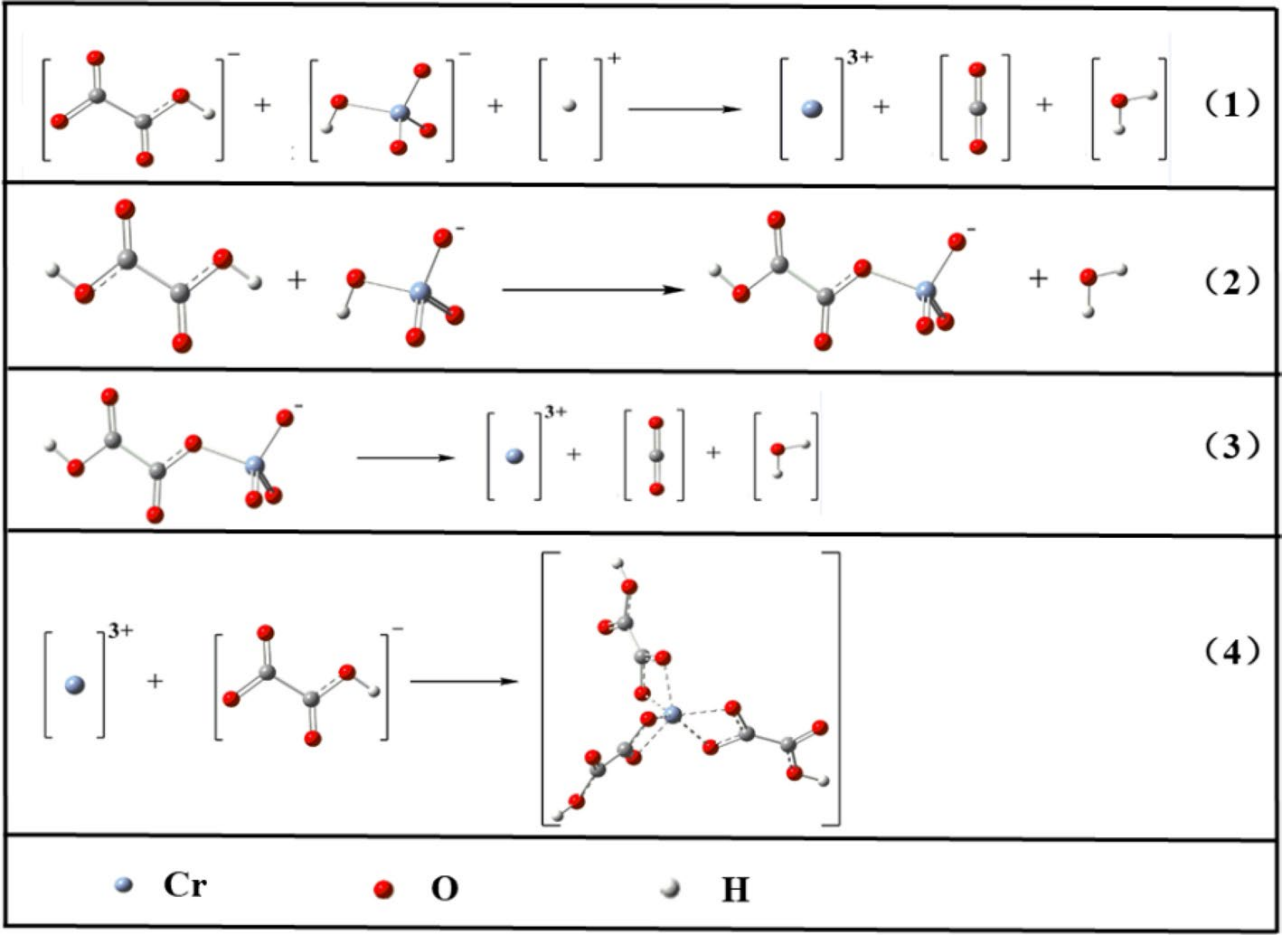
Reaction mechanism for reduction of Cr(VI) with oxalic acid.

During the Cr(VI) reduction process, the predominant Cr(VI) species was HCrO_4_^−^ and the reduction process was reacted following Eq. (1) showed in Fig. 2. The Cr(VI) was reduced to Cr^3+^ and H_2_C_2_O_4_ was oxidized to CO_2_. The reaction process could be divided into two parts: (I) The formation of ester-like compounds between HCrO_4_^−^ and H_2_C_2_O_4_ following Eq. (2). (II) The electron migration between the inner of ester-like compounds following Eq. (3)^21^.

### Reduction process

During the reduction process, the effect of dosage of oxalic acid, reaction temperature, dosage of sulfuric acid and reaction time on the reduction efficiency of Cr(VI) were investigated. The residual concentration of Cr(VI) was measured by ICP-OES and the results were shown in Table 1.

**Table 1:** Residual concentration of Cr(VI) measured by ICP-OES in various experimental conditions (mg/L).

n(O)/n(Cr)	n = 1.5	n = 3.0	n = 4.5	n = 6.0	
t	Cr(VI)	Cr(VI)	Cr(VI)	Cr(VI)	
**Effect of dosage of oxalic acid n(O)/n(Cr)**					
5	899.44	580.89	393.50	168.64	
10	824.49	468.45	262.34	74.95	
15	805.74	430.98	206.12	37.48	
20	787.01	412.24	149.91	18.74	
25	787.01	412.24	149.91	9.37	

T	30 °C	40 °C	50 °C	60 °C	70 °C
t	Cr(VI)	Cr(VI)	Cr(VI)	Cr(VI)	168.65
**Effect of reaction temperature (T)**					
5	562.15	430.98	393.50	262.34	74.95
10	430.98	318.55	224.86	112.43	37.48
15	337.29	224.86	149.90	56.21	18.74
20	262.33	168.65	112.43	37.48	9.37
25	224.86	131.17	74.95	18.74	–
30	187.38	93.69	56.21	9.37	–
35	149.91	74.95	37.48	–	–
40	112.43	56.12	18.74	–	–
45	93.69	37.48	9.37	–	–
50	74.95	18.74	–	–	–
55	56.21	9.37	–	–	–
60	37.48	–	–	–	–
65	18.74	–	–	–	–
70	9.37	–	–	–	–

n(O)/n(Cr) = 1.5	0 g/L	100 g/L	200 g/L	300 g/L	
t	Cr(VI)	Cr(VI)	Cr(VI)	Cr(VI)	
**Effect of dosage of sulfuric acid**					
5	899.44	693.32	618.36	599.63	
10	824.49	599.63	580.89	562.15	
15	805.74	543.41	505.94	487.19	
20	787.01	505.94	449.72	468.45	
25	787.01	468.46	430.98	449.72	
30	787.01	449.72	430.98	430.98	
35	787.01	449.72	430.98	430.98	

n(O)/n(Cr) = 3.0	0 g/L	100 g/L	200 g/L	300 g/L	
t	Cr(VI)	Cr(VI)	Cr(VI)	Cr(VI)	
5	580.89	374.77	318.55	299.81	
10	468.45	224.86	206.12	168.65	
15	430.98	149.91	131.17	131.17	
20	412.24	112.43	74.95	74.95	
25	412.24	74.95	56.21	56.21	
30	412.24	56.21	37.48	37.48	
35	412.24	37.48	18.74	18.74	
40	412.24	18.74	9.37	9.37	
45	412.24	9.37	–	–	

n(O)/n(Cr) = 4.5	0 g/L	100 g/L	200 g/L	300 g/L	
5	393.50	112.43	112.43	112.43	
10	262.34	37.48	37.48	37.48	
15	206.12	18.74	18.74	18.74	
20	149.91	9.73	9.73	9.73	

n(O)/n(Cr) = 6.0	0 g/L	100 g/L	200 g/L	300 g/L	
5	168.64	37.48	37.48	37.48	
10	74.95	9.37	9.37	9.37	
15	37.48	–	–	–	
20	18.74	–	–	–	
25	9.37	–	–	–	

**Figure 3:**
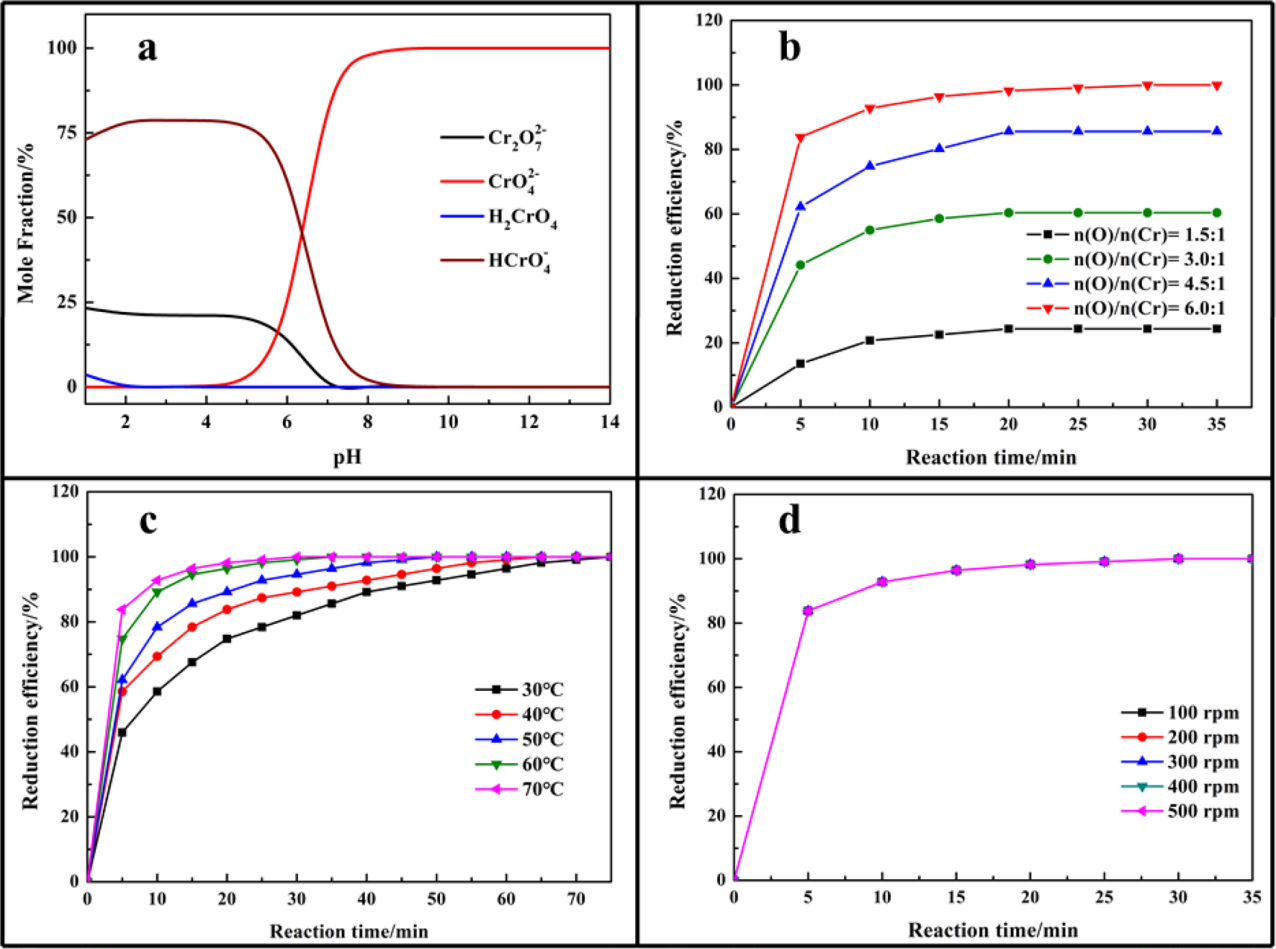
Effect of parameters on reduction efficiency of Cr(VI) (**a**) mole fraction of Cr(VI) species at various pH; (**b**) dosage of oxalic acid; (**c**) reaction temperature; (**d**) stirring rate.

The dosage of oxalic acid played an important role during the reduction process as it was the main reaction reagent. Some experiments were conducted to investigate the effect of the dosage of oxalic acid (n(O)/n(Cr)) on the reduction efficiency of Cr(VI) at reaction temperature of 70 °C with 500 rpm. The results shown in Fig. 3b indicating that reduction efficiency was increased with the increase of dosage of oxalic acid. The reduction efficiency was increased from 24.3 to 99.9% as dosage of oxalic acid increased from n(O)/n(Cr) = 1.5 to n(O)/n(Cr) = 6.0. At the beginning of the reduction process, the reduction efficiency of Cr(VI) was high due to the high concentration of Cr(VI) and oxalic acid and fast reaction rate. Along with the reduction process, the increasing trend of reduction efficiency of Cr(VI) became smooth due to the formation of a soluble Cr(III)-organic products, which formed by Cr^3+^ and oxalate (Eq. (4))^21^. In order to enhance the reduction process of Cr(VI), the high dosage of oxalic acid should be added as there was no enough oxalate to reduce Cr(VI) at a lower dosage of oxalic acid. Thus, the n(O)/n(Cr) = 6.0 was selected as an optimal condition in further experiments.

The effect of reaction temperature on the reduction efficiency of Cr(VI) was investigated under the standard conditions: initial concentration of Cr(VI) of 1000 mg/L, n(O)/n(Cr) = 6.0, and stirring rate at 500 rpm. It could be seen from Fig. 3c that the reduction efficiency of Cr(VI) could up to 100% at all reaction temperatures with enough reaction time, and it was easily achieved at a higher reaction temperature in low reaction time, which was partially consistent with a recent study. Higher temperature could increase the activity of atoms and molecules, enforced the reaction intensity, and promoted the reduction process of Cr(VI)^12,13,33^. Meanwhile, high reaction temperature could destroy the stable complex compound and release oxalate, which facilitated the reduction of Cr(VI), thus, the reduction efficiency of Cr(VI) was increased with the increase of reaction temperature. Therefore, the reaction temperature of 70 °C was selected as an optimal condition for further experiments.

Figure 3d summarized the effect of stirring rate on the reduction efficiency of Cr(VI) at reaction temperature of 70 °C, n(O)/n(Cr) = 6.0, and it showed that the reduction efficiency of Cr(VI) was all the same as stirring rate ranged from 100 to 500 rpm.

**Figure 4:**
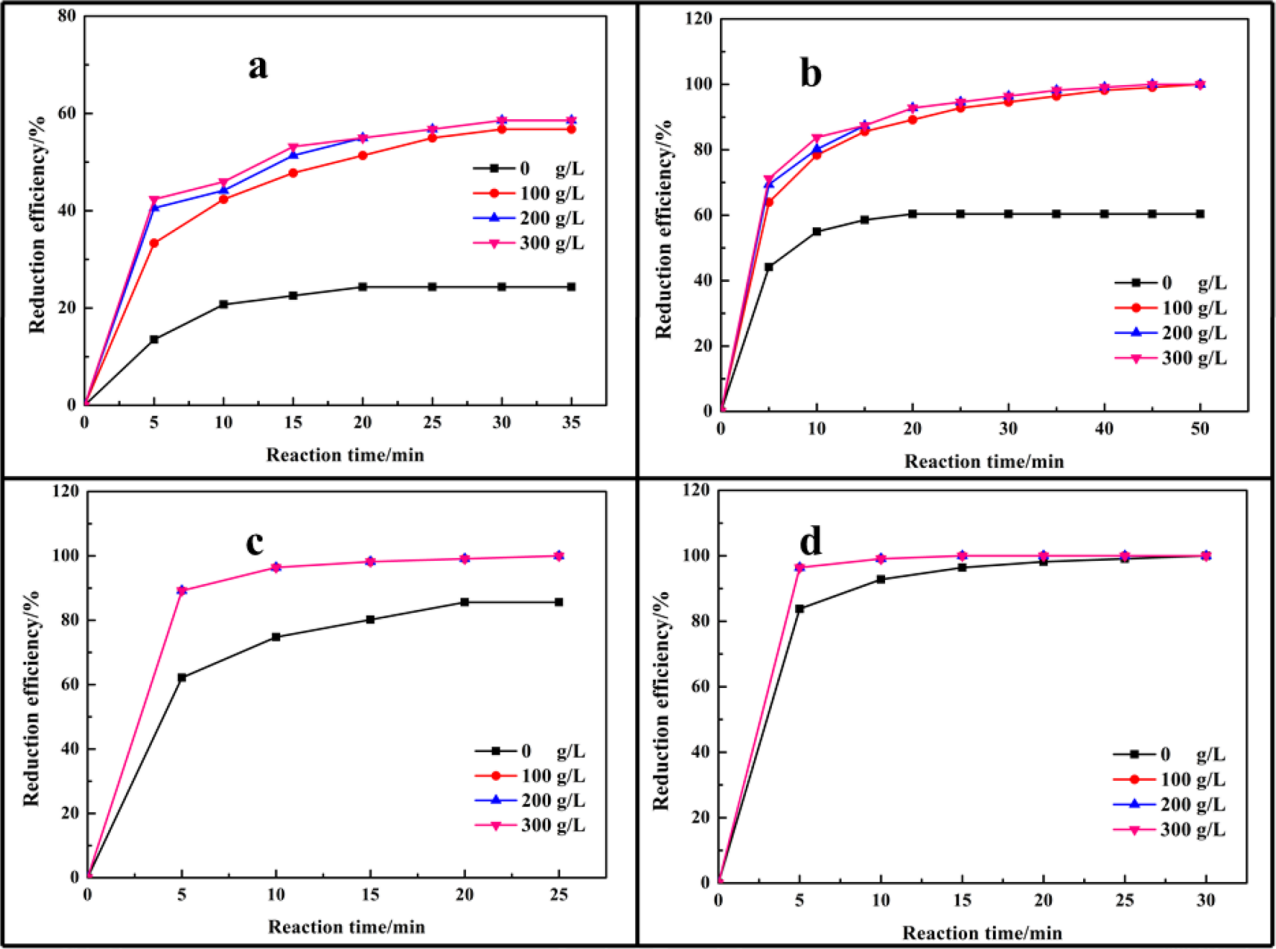
Effect of H_2_SO_4_ concentration on the reduction efficiency of Cr(VI) at various dosage of oxalic acid (**a**) n(O)/n(Cr) = 1.5:1; (**b**) n(O)/n(Cr) = 3.0:1; (**c**) n(O)/n(Cr) = 4.5:1; (**d**) n(O)/n(Cr) = 6.0:1.

The reduction of Cr(VI) to Cr(III) with oxalic acid could be favoured in the acid condition according to Eq. (1). The Cr(VI) reduction process with oxalic acid was investigated at concentration of H_2_SO_4_ ranged from 0 to 300 g/L in this study. Figure 4 displayed that the addition of H_2_SO_4_ could facilitate Cr(VI) reduction process. Theoretically, HCrO_4_^−^ was the predominant Cr(VI) species at 0.8 < pH < 6.8, and CrO_4_^2−^ was major species at pH > 6.8 according to the results showed in Fig. 3a, which measured by software Visual MINTEQ^34^, while HCrO_4_^−^ was easier reduced into Cr(III) than CrO_4_^2−^ as HCrO_4_^−^ possessed a higher oxidation potential (E^0^(HCrO_4_^−^/Cr^3+^) = 1.35 V, E^0^(CrO_4_^2−^/Cr^3+^) = 0.56 V). In the high acidic medium, the complex compound was not stable and released oxalate, which facilitated the reduction of Cr(VI), thus, the reduction efficiency of Cr(VI) was increased with the addition of H_2_SO_4_. Other way, the addition of H_2_SO_4_ could improve the reduction efficiency of Cr(VI), the concentration of H_2_SO_4_ had no obvious effect on the reduction efficiency at a high dosage of oxalic acid as the oxalate was enough.

### Kinetics analysis

The reduction behavior of Cr(VI) could be expressed by the pseudo-first-order equation as described as Eq. (5)^35–37^.

5 $$ \upsilon = \frac{dC}{{dt}} = - K_{obs} C $$

Integrate.

6 $$ - LnC = K_{obs} t - LnC_{0} $$

where *v,* is the reduction rate of Cr(VI), *C*, is the concentration of Cr(VI), *C*_0_, is the initial concentration of Cr(VI), *K*_*obs*_, is the reaction constant.

**Figure 5:**
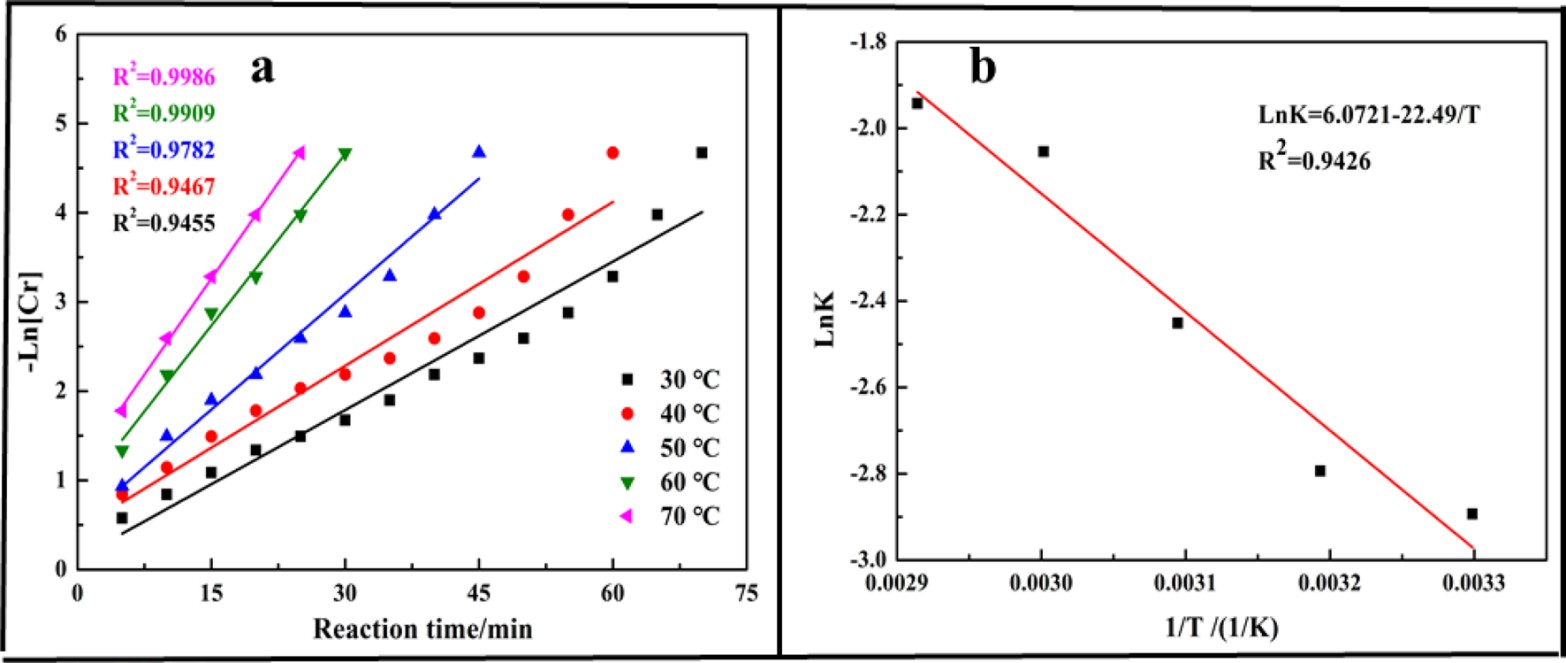
Kinetics plots: (**a**) Plot of reduction kinetics at various reaction temperatures; (**b**) natural logarithm of reaction rate constant versus reciprocal temperature.

The experimental data were fitted with Eq. (6) and the results shown in Fig. 5a indicating that the data were fitted well with the kinetics model as the coefficient (*R*^2^) all closely to 1, in other words, the reduction kinetics behavior of Cr(VI) was followed the pseudo-first-order model equation. The Arrhenius Equation (Eq. (7)) was applied to measure the relationship between *K*_*obs*_ and reaction temperature (T) and specific apparent activation energy. The result shown in Fig. 5b showed the simulated Arrhenius equation and the *Ea* was calculated as 22.49 kJ/mol, which was much larger than the apparent energy calculated for electrochemical reduction (4.74 kJ/mol)^12^. It meant that the reduction process by oxalic acid was harder than electrochemical reduction, while the reduction efficiency was much more efficient ((99.9% for reduction with oxalic acid and 86.45% for electrochemical reduction).

7 $$ \ln {\text{K}}_{{{\text{obs}}}} = \ln {\text{A}} - {\text{Ea}}/{\text{RT}} $$

where *Ea*, is the apparent activation energy, *A*, is the pre-exponential factor, and *R*, is the molar gas constant, *K*, is the reduction rate constant at different reaction temperatures.

## Conclusions

In this paper, the direct reduction process of Cr(VI) with oxalic acid was conducted. The following conclusions could be obtained:

1. The Cr(VI) was easily reduced by oxalic acid at high reaction temperature and a high dosage of oxalic acid in acidic medium. Nearly 99.9% of Cr(VI) was reduced at n(oxalic acid)/n(Cr) = 6.0 and reaction temperature of 70 °C. The dosage of acid concentration and stirring rate had no obvious effect on Cr(VI) reduction process.
2. The reduced product, Cr(III), was easily generated stable complex compounds (Cr(HC_2_O_4_)_3_) with oxalate, which displayed a negative effect on the reduction process. The high reaction temperature and high acidic medium could destroy the stable structure of a complex compound to release oxalate, and facilitate the reduction of Cr(VI).

## Materials and methods

### Materials

Potassium dichromate (K_2_Cr_2_O_7_), sulfate acid (H_2_SO_4_), and oxalic acid (H_2_C_2_O_4_·2H_2_O) were purchased from Kelong Co., Ltd, Chengdu, China. All chemicals were used as received without further purification. All solutions were prepared with deionized water with a resistivity greater than 18 MΩ/cm (HMC-WS10).

### Experimental procedure

All the experiments were carried out in a 250 mL beaker fixed in a thermostatic water bath with a temperature precision of ± 0.1 °C^12,13^. In the batch experiments, a volume of 100 mL solution contained 1000 mg/L Cr(VI) was prepared by dissolving K_2_Cr_2_O_7_ in deionized water, then the oxalic acid was added into the solution when the Cr(VI) solution heated to a predetermined temperature. During the experiments, the samples were collected at different intervals (5 min), and analyzed for residual concentration of Cr(VI) in the solution^12,13^. The reduction efficiency (η) of Cr(VI) was calculated as Eq. (8):

8 $$ \eta = \frac{{C{}_{0} - C{}_{t}}}{{C{}_{0}}} \times 100\% $$

where *C*_0_, is the initial concentration of Cr(VI) in the solution, mg/L; *Ct*, is the concentration of Cr(VI) in the solution at reaction time of t, mg/L.
